# Detrimental role for CCAAT/enhancer binding protein δ in blood-borne brain infection

**DOI:** 10.1186/s12879-016-1963-7

**Published:** 2016-11-11

**Authors:** JanWillem Duitman, Mercedes Valls Serón, JooYeon Engelen-Lee, Matthijs C. Brouwer, C. Arnold Spek, Diederik van de Beek

**Affiliations:** 1 Center for Experimental and Molecular Medicine (CEMM) Academic Medical Center, University of Amsterdam, P.O. Box 22660, 1100DD Amsterdam, The Netherlands; 2 Department of Neurology, Academic Medical Center, 1100DD Amsterdam, The Netherlands; 3 Neuroinfection and Inflammation, Amsterdam Neuroscience, Amsterdam, The Netherlands

**Keywords:** CAAT/enhancer-binding protein δ, C/EBPδ, Blood-borne brain infection, *Streptococcus pneumoniae*, Experimental meningitis

## Abstract

**Background:**

The most frequent pathogen that causes bacterial meningitis is the Gram-positive bacterium *Streptococcus (S.) pneumoniae*. CCAAT/enhancer binding protein δ is a transcription factor that has recently been hypothesized to play a detrimental role in outcome of meningitis caused by *S. pneumoniae*. Here, we studied the role of C/EBPδ prior to the development of pneumococcal meningitis.

**Methods:**

Wild-type and C/EBPδ-deficient mice (C/EBPδ^−/−^) were intraveneously infected with *S. pneumoniae* and sacrificed after 24 or 48 h. *cebp*δ expression, bacterial loads, inflammatory response and pathology in the brain were assessed.

**Results:**

*S. pneumoniae* induces *cebp*δ expression in the brain during blood-borne brain infection. In comparison to wild-type mice, C/EBPδ^−/−^ animals showed decreased bacterial loads in blood and brain 48 h after inoculation. In the blood compartment, the host inflammatory response was significantly lower upon infection in C/EBPδ^−/−^ mice as compared to wild-type mice.

**Conclusion:**

C/EBPδ facilitates bacterial dissemination to the brain and enhances the immune response in the blood compartment. Our study suggests that C/EBPδ plays a detrimental role during the initial development of blood-borne brain infection.

## Background

The Gram positive bacterium *Streptococcus (S.) pneumoniae* is a common colonizer of the respiratory tract [1]. *S. pneumoniae* can however become invasive and may spread from the upper respiratory tract to other organs, leading to life-threathening infections such as pneumonia, sepsis, or meningitis [2]. Meningitis is a disease of the central nervous system characterized by inflammation of the protective membranes covering the brain and spinal cord [3]. *S. pneumoniae* is the most common etiological agent of bacterial meningitis and causes 70 % of cases [4–6]. Despite the availability of effective antibiotic treatments and vaccination programs [7, 8], bacterial meningitis still has a high mortality rate in adult patients and almost half of survivors suffer from neurological sequelae (e.g., learning, hearing, and memory impartment, seizures, and motor deficits) due to permanent brain damage [6, 9–15]. Consequently, it is essential to improve existing therapies for meningitis through improving our understanding of the underlying pathophysiology.

CCAAT/enhancer binding protein (C/EBP) δ is a member of the C/EBP family of transcription factors that currently is composed of 6 different unique members (C/EBPα, C/EBPβ, C/EBPδ, C/EBPγ, C/EBPε and C/EBPζ) [16, 17]. C/EBPδ is generally accepted to act as a pro-inflammatory transcription factor, and was found to be essential in Fcγ receptor-mediated inflammatory cytokine and chemokine production. C/EBPδ deficient macrophages failed to induce a full tumour-necrosis factor (TNF)-α, macrophage inflammatory protein (MIP)-2 and MIP-1α response induced by IgG Immune complexes [18]. Moreover, low dose lipopolysaccharide (LPS) stimulation of macrophages induces C/EBPδ expression, leading to higher interleukin (IL)-6, Monocyte Chemoattractant Protein (MCP)-1 and endothelin-1 levels [19]. C/EBPδ also potentiates IL-6 expression in macrophages upon high dose LPS stimulation [20]. Recently, C/EBPδ was shown to play a pivotal role in the host response to gram-positive *S. pneumoniae* infections including pneumonia and meningitis [21, 22]. During pneumococcal pneumonia, C/EBPδ exaggerates bacterial dissemination and wild-type mice succumb earlier to the disease as compared to C/EBPδ^−/−^ mice whereas in pneumococcal meningitis increased C/EBPδ expression in the brain was associated with increased bacterial growth, dissemination and the inflammatory response.

Most in vivo models that study the pathophysiology of bacterial meningitis involve the direct injection of pneumococci into the brain of mice or rats [23] and therefore aim to study host-pathogen interactions once infection is established in the meninges. The aim of the current study was to investigate the role of C/EBPδ prior to the onset of meningitis. Since an important route of central neurvous system (CNS) infection by bacterial pathogens is via the blood stream, we challenged wild-type and C/EBPδ^−/−^ mice with *S. pneumoniae* through intravenous injections. We show that *S. pneumoniae* induces C/EBPδ expression in the brain in blood-borne brain infection. Moreover, C/EBPδ^−/−^ animals showed decreased bacterial loads in blood and brain 48 h after inoculation. The reduced bacterial dissemination in the brain did however not result in a lower inflammatory response or reduced histopathology in the brain of C/EBPδ^−/−^ mice. Thus, our study suggests that C/EBPδ^−/−^ modifies bacterial dissemination in blood-borne brain infection.

## Methods

### Animals

C/EBPδ^−/−^ mice, generated as described previously [24], were backcrossed at least 10 times to a C57BL/6 background. Wild-type mice were purchased from Charles River (Maastricht, the Netherlands). 8- to 12-week-old male or female animals were maintained at the animal facility of the Academic Medical Center (University of Amsterdam) with free access to food and water. All animal experiments were approved by the Institutional Animal Care and Use Committee of the Academic Medical Center, University of Amsterdam.

### Sepsis infection model

Wild-type and C/EBPδ^−/−^ mice (*n* = 30 per group) were inoculated into the tail vein with 5 × 10^5^ CFU of *S. pneumoniae* serotype 3, American Type Culture Collection 65303 (in 200 μl saline) as previously described [25, 26]. Control animals (*n* = 6) received saline only. At 24 h and 48 h after inoculation, organs were collected and homogenised as described previously [27].

### Determination of cytokines and chemokines

TNF- α, IL-6, Interferon (IFN)-γ and MCP-1 levels were determined using a cytometric bead array multiplex assay (BD Bioscience, San Jose, CA, USA) as described previously [27].

### Real-time PCR

Total RNA was extracted from murine brain homogenates using TriPure reagent (Sigma-Aldrich, St-Louis, MO, USA). For complementary DNA (cDNA) synthesis, RNA was treated with RQ1 RNase-free DNase (Promega, Leiden, the Netherlands) and reverse transcribed with SuperScript II Reverse Transcriptase and random hexamers (Life Technologies, Bleiswijk, the Netherlands). The real-time polymerase chain reaction (RT-PCR) was performed on a Bio-Rad MyiQ Single-Color RT-PCR Detection System using the Bio-Rad iQ SYBR Green Supermix (Bio-Rad Laboratories, Hercules, CA, USA). The *c/ebpδ* and Non-POU-domain containing octamer binding protein (NoNo, housekeeping gene), primers were described previously [22]. The C/EBPδ expression levels were normalized to the NoNo reference gene.

A negative control without the Reverse Transcriptase was also used.

### Statistical analysis

All data are expressed as means ± SEM. Differences between groups were analyzed by *t*-test and when necessary corrected for nonparametric values by Mann-Whitney *U* test. Differences in bacteremic brains were analysed by Fisher’s exact test. Correlation was analysed by correlation analysis. Analyses were performed using GraphPad Prism version 6.0 (GraphPad Software, San Diego, CA, USA) or R [28]. Statistically significant differences were considered with a *p* value less than 0.05.

## Results

### C/EBPδ expression is increased in brain during pneumococcal sepsis

**Fig. 1:**
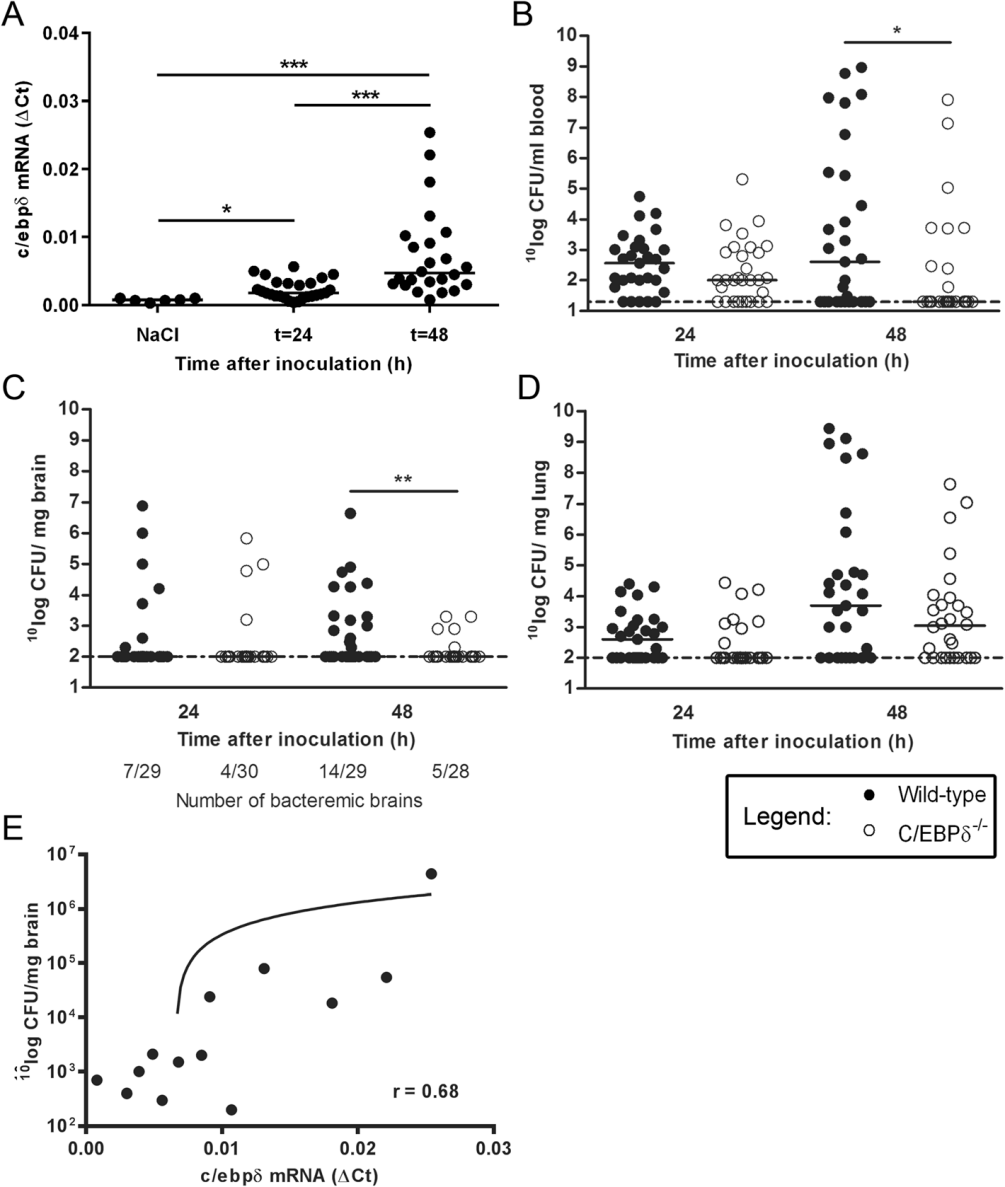
C/EBPδ is detrimental for the blood-brain barrier upon intravenous pneumococcal infection. *c/ebpδ* mRNA (**a**) in wild-type brain tissue at different time points after intravenous inoculation with *S. pneumoniae.* Bacterial outgrowth in whole blood (**b**), brain (**c**) and lung (**d**) of wild-type and C/EBPδ^−/−^ 24 and 48 h after infection. Correlation analysis of *cebpd* mRNA expression and bacterial loads in the brain (**e**). Data are expressed as scatter dot plots with the median (*n* = 6 for controls; *n* = 30 at both t = 24 and t = 48 h post infection). * *P* < 0.05, ** *P* < 0.01, *** *P* < 0.001

To determine C/EBPδ expression in the brain during sepsis caused by *S. pneumoniae*, we measured *c/ebpδ* mRNA levels in brain tissue from wild-type mice inoculated with 5 × 10^5^ collony forming units (CFU). As shown in Fig. 1a, *c/ebpδ* mRNA levels were low in brain of uninfected mice but significantly increased at 24 h (approximately 3-fold) and 48 h (approximately 10-fold) after *S. pneumoniae* inoculation.

### C/EBPδ aggravates bacterial dissemination from the blood to the brain

In order to investigate the role of C/EBPδ in the development of meningitis upon pneumococcal sepsis, wild-type and C/EBPδ^−/−^ mice were intravenously inoculated with *S. pneumoniae*. Over time the bacterial loads increased in wild-type mice in blood, brain and lung. After 48 h, C/EBPδ^−/−^ mice had significant lower bacterial count in the blood as compared to wild-type (Fig. 1b). As shown in Fig. 1c, C/EBPδ^−/−^ mice also presented lower bacterial counts in the brain as compared to wild-type mice 48 h after inoculation. Notably, the number of mice with bacteremic brains was increased in wild-type mice at 48 h (14/29 versus 5/28, p = 0.02, odds ratio [OR] 4.2, 95 % confidence interval [CI] 1.13–18.1), whereas the number was similar in C/EBPδ^−/−^ mice at 24 h (7/29 versus 4/30, *p* = 0.33, OR 2.04, CI 0.45–10.83). No difference was observed in bacterial counts in the lungs of wild-type and C/EBPδ^−/−^ mice (Fig. 1d). Correlation analysis of *cebpd* mRNA expression and bacterial loads in the brain shows that C/EBPδ expression is positively and significantly correlated with the bacterial burden (*p* = 0.01, Spearman r = 0.68; Fig. 1e), suggesting that the increase in C/EBPδ expression leads to bacterial dissemination.

### C/EBPδ does not affect the inflammatory response in brain during pneumococcal sepsis

**Fig. 2:**
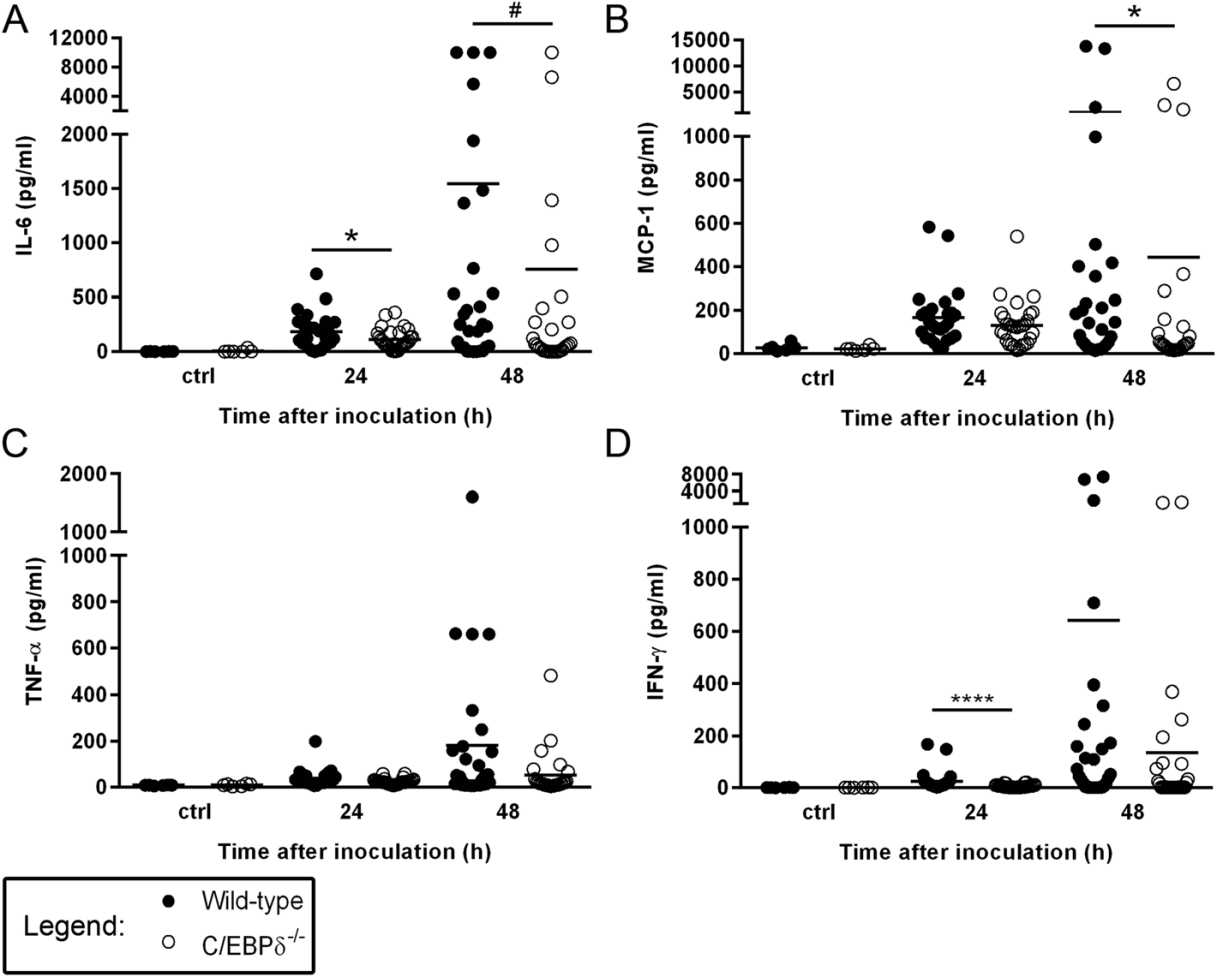
C/EBPδ affects the inflammatory response in plasma in pneumococcal blood-borne brain infection. Levels of IL-6 (**a**), MCP-1 (**b**), TNF-α (**c**) and IFN-γ (**d**) in plasma of Wild-type and C/EBPδ ^−/−^ mice in *S. pneumoniae* blood-borne brain infection. Data are expressed as scatter dot plots with the median (*n* = 6 for controls; *n* = 30 at both t = 24 and t = 48 h post infection). *# P = 0.054*, * *P* < 0.05, **** *P* < 0.0001

To determine whether C/EBPδ affects the inflammatory response in blood and brain during pneumococcal sepsis we measured different cytokine and chemokine levels in plasma and brain homogenates. As shown in Fig. 2, the host response as measured by cytokine and chemokine production in plasma was increased over time in both wild-type and C/EBPδ^−/−^ mice. IL-6 levels (Fig. 2a) were significantly lower at both 24 and 48 h post inoculation in C/EBPδ^−/−^ mice as compared to wild-type mice. IFN-γ (Fig. 2d) and MCP-1 (Fig. 2b) levels were significantly lower at 24 (IFN-γ) or 48 (MCP-1) hours post inoculation. No differences in TNF-α levels were observed between wild-type and C/EBPδ^−/−^ mice (Fig. 2c). In brain, all measured cytokine and chemokine levels were very low (data not shown) and histological analysis of the brains did also not show clear signs of meningitis (data not shown).

## Discussion

In the present study, we demonstrate that C/EBPδ plays a detrimental role during *S. pneumoniae* sepsis-induced meningitis. We show that C/EBPδ expression in the brain is induced after an intravenous challenge with *S. pneumoniae*, and that it aggravates bacterial dissemination from the blood to the brain thereby driving the progression towards meningitis. This notion is strengthened by the positive correlation between C/EBPδ gene expression levels and bacterial counts in the brain 48 h post challenge.

Several studies have implicated C/EBPδ as regulator of proinflammatory cytokine expression [29], which are in line with our finding that 24 h after inoculation, IL-6 and INF-γ levels in plasma were significantly lower in the absence of C/EBPδ; and at 48 h post challenge, IL-6 and MCP-1 levels were lower in C/EBPδ^−/−^ mice. However, we were not able to detect differences in cytokine levels between groups in the brain compartment since pneumococcal sepsis only caused a very modest inflammatory response in the brain, as reflected by low inflammatory cytokine levels. In accordance with the inflammatory cytokine profile in brain, the absence of brain histopathological meningitis traits, even at 48 h, indicates that the experimental model is merely suitable to study the initial process of the development of pneumococcal meningits. Because the mice eventually will start to clear the bacteria shortly after 48 h post inoculation, the sepsis model used is not suitable to study prolonged time points beyond 48 h which is a limitation of our study.

In addition to the difference in bacterial loads in the brain we did not observe a difference in dissemination towards the lungs. This is in line with our previous study [21] in which we specifically studied the role of C/EBPδ in *S. pneumoniae*-induced pulmonary infection. In the previous study we did not observe a difference in bacterial loads in the blood of wildtype and C/EBPδ^−/−^ mice, which is in contrast with the current study where we did observe a difference in bacterial loads in the blood at 48 h post inoculation. The discrepancy between the two studies may be caused by the number of mice included. The number of mice included in the current study is approximately four times higher (8 versus 30 for the previous study and the current study respectively) which may have increased the power of the statistical analysis leading to a significant difference in the current study. More importantly however, the lack of a significant difference in dissemination towards the lungs suggests that the observed difference in bacterial loads in the brain between wildtype and C/EBPδ^−/−^ mice in the current study is not merely a reflection of the bacterial loads in the blood. Therefore we conclude that C/EBPδ plays a specific role in the dissemination of *S. pneumoniae* towards the brain.

Previously we have shown that upon intracisternal inoculation of pneumococci, C/EBPδ^−/−^ mice showed a decrease in bacterial outgrowth and inflammatory response in the brain as compared to wild-type mice [22]. Here we show that C/EBPδ^−/−^ mice have limited bacterial dissemination towards the brain upon intravenous inoculation of pneumococci. Taken together, these results show that C/EBPδ plays a dual and detrimental role during both the establishment and disease progression of pneumococcal meningitis. It can therefore be speculated that inhibition of C/EBPδ may reduce bacterial dissemination during both the establishment and subsequent progression of pneumococcal meningitis. However, further studies should elucidate the role of C/EBPδ as potential target for novel therapeutic interventions during meningitis.

## Conclusions

Our results show that C/EBPδ expression in the brain increased in response to systemic *S. pneumoniae* infection, that C/EBPδ^−/−^ mice presented reduced bacterial dissemination to the brain and displayed a lower inflammatory response in plasma as measured by MCP-1 and IL-6. Overall, our results show that C/EBPδ plays a detrimental role during the initial development of meningitis caused by sepsis.

## Abbreviations

C/EBP: CCAAT/enhancer binding protein
cDNA: complementary DNA
CFU: Collony forming units
CI: Confidence interval
CNS: Central nervous system
IFN: Interferon
IL: Interleukin
LPS: Lipopolysaccharide
MCP: Monocyte chemoattractant protein
MIP: Macrophage inflammatory protein
NoNo: Non-POU-domain containing octamer binding protein
OR: Odds ratio
RT-PCR: Real-time polymerase chain reaction
*S. pneumoniae*: *Streptococcus pneumoniae*
TNF: Tumour-necrosis factor
